# l-Cystine-Crosslinked Polypeptide Nanogel as a Reduction-Responsive Excipient for Prostate Cancer Chemotherapy

**DOI:** 10.3390/polym8020036

**Published:** 2016-01-29

**Authors:** Liang He, Di Li, Zhongtang Wang, Weiguo Xu, Jixue Wang, Hui Guo, Chunxi Wang, Jianxun Ding

**Affiliations:** 1 Department of Urology, the First Hospital of Jilin University, Changchun 130021, China; lianghe9278@126.com (L.H.); wangjx@ciac.ac.cn (J.W.); 18443154199@163.com (H.G.); 2 Key Laboratory of Polymer Ecomaterials, Changchun Institute of Applied Chemistry, Chinese Academy of Sciences, Changchun 130022, China; lidi@ciac.ac.cn (D.L.); wgxu@ciac.ac.cn (W.X.); 3 Department of Radiation Oncology, Shandong Cancer Hospital and Institute, Jinan 250117, China

**Keywords:** chemotherapy, l-cystine, polypeptide nanogel, prostate cancer, reduction-responsiveness, smart drug delivery

## Abstract

Smart polymer nanogel-assisted drug delivery systems have attracted more and more attention in cancer chemotherapy because of their well-defined morphologies and pleiotropic functions in recent years. In this work, an l-cystine-crosslinked reduction-responsive polypeptide nanogel of methoxy poly(ethylene glycol)-poly(l-phenylalanine-*co*-l-cystine) (mPEG-P(LP-*co*-LC)) was employed as a smart excipient for RM-1 prostate cancer (PCa) chemotherapy. Doxorubicin (DOX), as a regular chemotherapy drug, was embedded in the nanogel. The loading nanogel marked as NG/DOX was shown to exhibit glutathione (GSH)-induced swelling and GSH-accelerated DOX release. Subsequently, NG/DOX showed efficient cellular uptake and proliferation inhibition. Furthermore, NG/DOX presented enhanced antitumor efficacy and security in an RM-1 PCa-grafted mouse model *in vivo*, indicating its great potential for clinical treatment.

## 1. Introduction

Prostate cancer (PCa) is the second most frequently diagnosed cancer in men worldwide, with 1.1 million new cases estimated to have occurred in 2012. PCa is also the most prevalent cancer among men in developing countries, and about two-thirds of all PCa cases occur among just 17% of the world’s male population [1]. Prostatectomy, radiotherapy, androgen deprivation therapy (ADT), and chemotherapy are the most important clinical treatments for PCa. However, prostatectomy just adapts to the patients with local PCa [2]; radiotherapy always causes unacceptable sexual toxicity [3]; and ADT treatment alone is useless for advanced PCa and always leads to castration-resistance [4]. Considering these disadvantages of surgery, radiotherapy, and endocrine treatment, chemotherapy should be the most important palliative treatment for PCa.

Regrettably, the traditional chemotherapy drugs, especially small-molecule ones (*i.e.*, doxorubicin (DOX)), have many drawbacks: short circulation time, low bioavailability, non-specific cytotoxicity, and drug resistance [5,6]. For these reasons, polymer nanoparticle-assisted chemotherapy formulations have gradually emerged in recent decades [7]. Polymer nanocarriers, such as polymer micelles [8,9,10], vesicles [11,12], and nanogels [13,14,15], play major roles in antitumor drug delivery because of their various merits, including prolonged blood circulation time, and improved intratumoral accumulation by the enhanced permeability and retention (EPR) effect, and/or active targeting. For PCa chemotherapy, the preclinical results revealed that polymer nanocarriers show great potential in improving the efficacy and reducing the side effects of traditional chemotherapy drugs, indicating their great promise for clinical use [16,17].

Among all the polymer nanoparticles, nanogels exhibit the greatest antitumor application prospects due to their excellent biocompatibility and biodegradability, easy modification, high drug loading capability, passive targetability, and so on. Needless to say, nanogels with tumor microenvironment-response characteristics have attracted tremendous fascination as excipients of antitumor pharmaceuticals [15]. The microenvironments of tumor tissue and cells are drastically different from those of normal ones, with low pH [18,19], hypoxia [20], and high level of glutathione (GSH) [14,21]. Plenty of stimuli-responsive polymer nanoparticles are designed to target the difference between extracellular and intracellular components.

**Figure 1:**
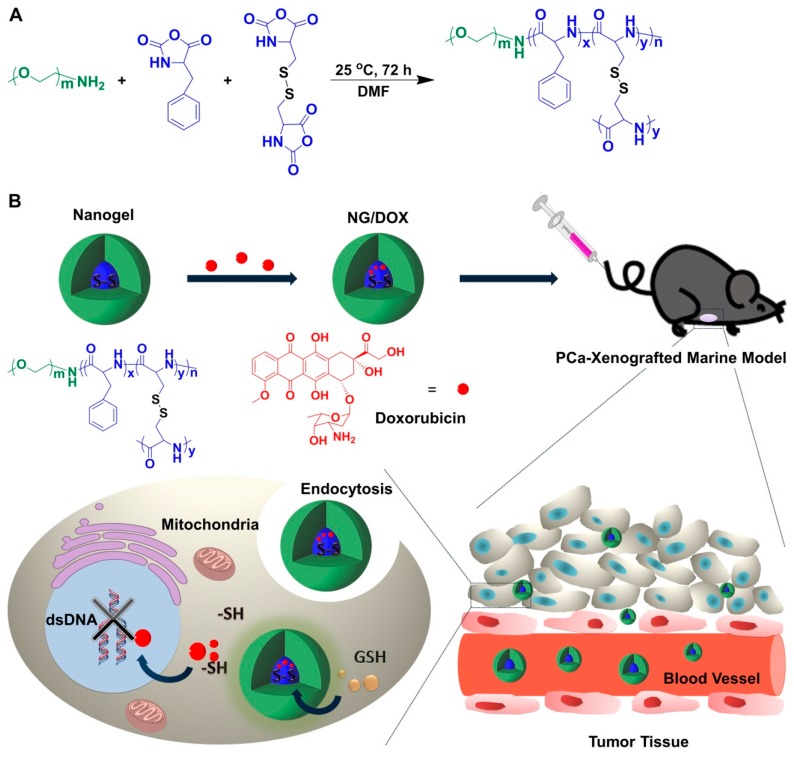
Synthesis pathway of mPEG-P(LP-*co*-LC)) nanogel (**A**). Schematic illustration for drug encapsulation, tail vein injection, *in vivo* circulation, intratumoral accumulation, endocytosis, and intracellular DOX release of l-cystine-crosslinked polypeptide nanogel (**B**).

In particular, there is always 50–1000 times the GSH concentration in tumor cells than that in the extracellular microenvironment [15]. The high level of GSH is of interest to the designer of polymer nanoparticle-based drug delivery platforms. In this aspect, the reduction-responsive nanogels have gradually become research hotspots in intracellular drug delivery [22]. As for PCa, the GSH level also shows great difference between extracellular and intracellular conditions. With the progression of PCa, the cellular redox status changed from more pro-oxidative to more anti-oxidative [23]. Meanwhile, the level of redox status is highly associated with the aggressive level of PCa [24]. The high level of intracellular GSH concentration guarantees the efficacy of reduction-responsive nanogels to be applied in PCa chemotherapy. In this work, as depicted in Figure 1A, a reduction-responsive methoxy poly(ethylene glycol)-poly(l-phenylalanine-*co*-l-cystine) (mPEG-P(LP-*co*-LC)) nanogel was synthesized *via* the one-step ring-opening polymerization (ROP) of l-phenylalanine *N*-carboxyanhydride (LP NCA) and l-cystine *N*-carboxyanhydride (LC NCA) with the amino-terminated mPEG (mPEG–NH_2_) as a macroinitiator according to the protocol reported in our previous studies [15,25,26]. DOX as a model antitumor drug was embedded into the core of nanogel to prepare a novel chemotherapy formulation (Figure 1B). The reduction-response characteristic-enhanced antitumor efficacy toward RM-1 murine PCa models *in vitro* and *in vivo*, and improved security *in vivo* were well confirmed. In brief, the reduction-responsive l-cystine-crosslinked polypeptide nanogel was shown to be a promising excipient of chemotherapy formulation with exceptional antitumor capability toward PCa, preclinically.

## 2. Experimental Section

### 2.1. Materials

As shown in Figure 1A, the reduction-responsive mPEG_113_-P(LP_6_-*co*-LC_4_) nanogel was synthesized through the simultaneous ROP of LP NCA and LC NCA following our previous proposal [15,25]. The subscript number represented the degree of polymerization (DP) of each component, which was calculated from elemental analysis. Doxorubicin hydrochloride (DOX·HCl) was purchased from Beijing Huafeng United Technology Co., Ltd. (Beijing, China). Glutathione (GSH) used for cell culture was purchased from Aladdin Reagent Co., Ltd. (Shanghai, China). dl-Dithiothreitol (DTT) was obtained from Adamas-Beta^®^ Co., Ltd. (Shanghai, China). Methyl thiazolyl tetrazolium (MTT) and 4′,6-diamidino-2-phenylindole (DAPI) were all sourced in Sigma-Aldrich (Shanghai, China). Clear 6-well and 96-well tissue culture polystyrene (TCP) plates were purchased from Corning Costar Co. (Cambridge, MA, USA). The deionized water was prepared through Milli-Q water purification equipment (Millipore Co., Billerica, MA, USA).

### 2.2. DOX Encapsulation

As mentioned in our previous works, DOX was embedded into nanogel through a sequential dispersion and dialysis approach [15,25,26]. In detail, nanogel (500.0 mg) was dispersed in 18.0 mL of *N*,*N*-dimethylformamide (DMF), and DOX·HCl (106.6 mg) was dissolved in 2.0 mL of DMF ether. Then the mixture of 18.0 mL of MilliQ water and 2.0 mL of phosphate-buffered saline (PBS, 0.01 M) was dropwise added into the above solution. The final solution was stirred for 12 h at room temperature, and then dialyzed against MilliQ water for 24 h (molecular weight cut-off (MWCO) = 3500 Da). The MilliQ water was replaced every 2 h. Subsequently, the DOX-loaded nanogel (referred as NG/DOX) was collected by lyophilisation after filtration.

The drug loading content (DLC) and drug loading efficiency (DLE) were further detected. In brief, 1.0 mg of NG/DOX was dissolved in 10.0 mL of DMF and stirred for 12 h at room temperature. Then, the DOX loaded in nanogel was detected by fluorescence spectroscopy on a Photon Technology International (PTI) Fluorescence Master System with Felix 4.1.0 software (PTI, Lawrenceville, NJ, USA; λ_ex_ = 480 nm and λ_em_ = 590 nm). The DLC and DLE of NG/DOX were calculated by Equations (1) and (2), respectively.

(1) $$\text{DLC}\;(\text{wt}\;\%)=\frac{\text{Weight of Drug in Nanogel}}{\text{Weight of Drug-Loaded Nanogel}}\times 100\%$$

(2) $$\text{DLE}\;(\text{wt}\;\%)=\frac{\text{Weight of Drug in Nanogel}}{\text{Total Weight of Feeding Drug}}\times 100\%$$

### 2.3. Characterizations

The microimage of laden nanogel was taken using a JEW-1011 transmission electron microscope (TEM, JEOL, Tokyo, Japan) with an accelerating voltage of 100 kV for the detection of morphology and apparent size. The nanoformulation size changes in normal PBS or PBS with 10.0 mM DTT were detected by dynamic laser scattering (DLS). The DLS tests were operated through a Wyatt QELS instrument (Wyatt Technology Corp., Santa Barbara, CA, USA).

### 2.4. In Vitro DOX Release

The *in vitro* drug release was studied using a dialysis method. Conventionally, 1.0 mg of freeze-dried NG/DOX was dissolved in 10.0 mL of PBS at pH 7.4 with (w GSH) or without 10.0 mM GSH (wo GSH), and then transferred into the end-sealed dialysis bag (MWCO = 3500 Da). Subsequently, the filled dialysis bag was put into 150 mL beaker, 100.0 mL of corresponding buffer was added rapidly, and the apparatus was finally placed into an oscillation box with continuous vibration of 70 revolutions per minute (rpm) at 37 °C to mimic the normal physiological microenvironment. At the predetermined time points, 2.0 mL of the external release medium in the beaker was taken out, and the equal volume of fresh medium was replenished into the container. The amount of released DOX was determined through standard curve method on a fluorescence spectrometer (λ_ex_ = 480 nm and λ_em_ = 590 nm).

### 2.5. Cell and Animal Proposals

RM-1 cells were cultured in complete high glucose Dulbecco’s modified Eagle’s medium (HG-DMEM) supplemented with 10% (*v*/*v*) fetal bovine serum (FBS), penicillin (50 IU·mL^−^^1^), and streptomycin (50 IU·mL^−^^1^) at 37 °C in 5% (*v*/*v*) carbon dioxide (CO_2_).

5-week-old male C57BL/6 mice weighting 21.7 ± 1.3 g were provided by the Charles River Laboratories (Beijing, China). All animal experiments were conducted in accordance with the Guidelines for Animal Care and Use of Jilin University.

### 2.6. In Vitro Cell Viability Assessment of NG/DOX

The cytotoxicity of free DOX and NG/DOX was measured toward RM-1 cells by a MTT assay. 1.0 × 10^4^ RM-1 cells were suspended in 200.0 μL of complete HG-DMEM, planted into each well of 96-well plates, incubated for 24 h, and then penetrated with 10.0 mM GSH for 2 h. The cells without the GSH pretreatment were used as control. After 2 h pretreatment, the culture medium was removed, the cells were washed by sterile PBS, and then 180.0 μL of fresh complete HG-DMEM was added. In addition, 20.0 μL of PBS containing free DOX and NG/DOX with concentrations from 0.08 to 10.0 μg·mL^−1^ were added to the experimental groups. Simultaneously, 20.0 μL of blank PBS was added to the control group. The cells were subjected to 20.0 μL of MTT at a concentration of 5.0 mg·mL^−1^ after being incubated for 24 or 48 h. After that, the cells were incubated for another 4 h in incubator. The culture medium was carefully removed, and 150.0 μL of dimethyl sulfoxide (DMSO) per well was added to dissolve the MTT formazan generated by live cells. Finally, the plates were shocked for 3 min, and the absorbance of the medium was detected at 490 nm by an ELx808 microplate reader (Bio-Tek^®^ Instruments, Inc., Winooski, VT, USA). The cell viability was calculated by Equation (3).

(3) $$\text{Cell Viability}\;(\%)=\frac{A_{\text{sample}}}{A_{\text{control}}}\times 100\%$$

### 2.7. Intracellular DOX Release

3 × 10^5^ of RM-1 cells suspended in 2.0 mL of HG-DMEM were seeded in each well of 6-well plates. After 24 h culture, the residual culture medium was removed and then 1.0 mL HG-DMEM with 10.0 mM GSH were added to the experimental groups for 2 h in order to improve the intracellular concentration of GSH, and the cells without the GSH pretreatment were considered as control. Then, the culture media were removed and filled with 2.0 mL of fresh HG-DMEM. Next, the cells of experimental group were co-incubated with free DOX or NG/DOX at a final DOX concentration of 5.0 μg·mL^−^^1^, and cells were co-incubated with PBS as the control group. After 2 h co-incubation, the cells were washed with PBS 5 times. After that, all cells were digested by 1.0 mL of trypsin per well and suspended in PBS. Then the harvested cells were centrifuged at 3000 rpm for 5 min. The supernatants were discarded, and the bottom cells were washed with PBS. Then the cells were resuspended in 400.0 μL of PBS, Data was analyzed by a flow cytometer (FCM; λ_ex_ = 488 nm; Guava EasyCyte™ technologies Inc., Hayward, CA, USA).

Similarly to the FCM detections, confocal laser scanning microscopy (CLSM) assays were performed to reveal the subcellular distribution of both DOX formulations. Typically, 1.5 × 10^5^ of RM-1 cells in 2.0 mL HG-DMEM were seeded on glass coverslips in a 6-well plate. After 24 h culture, 2.0 mL of HG-DMEM was replaced by 1.0 mL of HG-DMEM with 10.0 mM GSH, and the cells cultured for another 2 h. The cells without the GSH pretreatment were considered as control. Then, the culture medium in 6-well plate was removed, and 2.0 mL of fresh HG-DMEM is replenished. Next, the cells were co-incubated with free DOX or NG/DOX at a final DOX concentration of 5.0 μg·mL^−^^1^. The cells on glass sheet were further incubated for 2 h and then washed with PBS 5 times. After that, cells were fixed with 4% (*w*/*v*) PBS-buffered paraformaldehyde for 20 min. After being washed for 5 times by PBS, the cells were added in 0.1% (*v*/*v*) Triton X-100 in PBS for 12 min at room temperature, and then washed as mentioned above. Finally, cell nuclei were dyed blue by DAPI for 5 min, and washed with PBS for another 5 times. The CLSM microimages were taken by a CLSM (LSM 780, Carl Zeiss, Jena, Germany).

### 2.8. In Vivo Antitumor Efficacy Assays

In order to determine the antitumor efficacies of free DOX and NG/DOX *in vivo*, 3.0 million RM-1 cells were suspended in 0.1 mL of saline, and then injected in the right flank of C57BL/6 mice subcutaneously. When the tumor weight reached 8.7 × 10^−2^ g, mice were randomly divided into 3 groups (*n* = 8 for each group): normal saline (NS) as control, free DOX, and NG/DOX groups. For both DOX formulations, the dose of DOX is 5.0 mg per kg body weight (mg (kg·BW)^−1^). The treatments were performed by injecting all the formulations into the tail vein on Day 2, 4, and 9 to mimic the clinical impulsive antitumor-chemotherapy. The tumor weight, body weight, and survival rate were detected each day for evaluate antitumor efficacy and drug security. During treatment, tumor weight was detected through Equation (4) [27]. Meanwhile, tumor index was evaluated through Equation (5) to exhibit the tumor inhibition efficacy.

(4) $$\text{Tumor Weight}\;(g)=\frac{L\times S^{2}}{2}$$

(5) $$\text{Tumor Index}\;(\%)=\frac{\text{Tumor Weight}}{\text{Body Weight}}\times 100\%$$

In Equation (4), *L* and *S* (cm) were the largest and smallest diameters of tumor, respectively. In Equation (5), tumor weight (g) was calculated through Equation (4), and body weight (g) was recorded every day.

### 2.9. Histopathological Assessments

The RM-1 PCa-grafted C57BL/6 mice were sacrificed by cervical dislocation four days after the last treatment. After that, the tumor and major organs (*i.e.*, heart, liver, spleen, lung, and kidney) were collected and infiltrated in 4% (*w*/*v*) PBS-buffered paraformaldehyde for 24 h. Subsequently, the samples were followed by dehydration, clearing, wax infiltration, and embedding. Finally, ~5 μm thick paraffin sections were prepared for H & E staining.

In addition, the sternums from PCa-grafted C57BL/6 mice were simultaneously collected and infiltrated in 10% (*v*/*v*) formic acid-formalin solution. Then, the sternum samples were decalcified, fixed, wax infiltrated, and embedded. ~5 μm paraffin sections for sternums were performed for H & E staining. After that, the bone marrow cell micronucleus rate (BMMR) of each sample was observed under microscope (Nikon Eclipse *Ti*, Optical Apparatus Co., Ardmore, PA, USA).

### 2.10. Statistical Analyses

All experiments were carried out at least three times. The data were expressed as mean ± standard deviation (SD). Statistical analysis was performed using SPSS 13.0 for Windows (SPSS Inc., Chicago, IL, USA), *p* < 0.05 was considered statistically significant, and *p* < 0.01 and *p* < 0.001 were considered highly significant, respectively.

## 3. Results and Discussion

### 3.1. Preparation and Characterizations of NG/DOX

In this work, the reduction-responsive nanogel was composed of a hydrophilic mPEG shell and hydrophobic disulfide-cross-linked P(LP-*co*-LC) core (Figure 1A) [25]. As shown in Figure 1B, DOX, one of the most common clinical broad-spectrum chemotherapy drugs, was loaded into the core of nanogel through dispersion technique. The DLC and DLE of NG/DOX were calculated to be 8.81 and 48.3 wt %, respectively.

**Figure 2:**
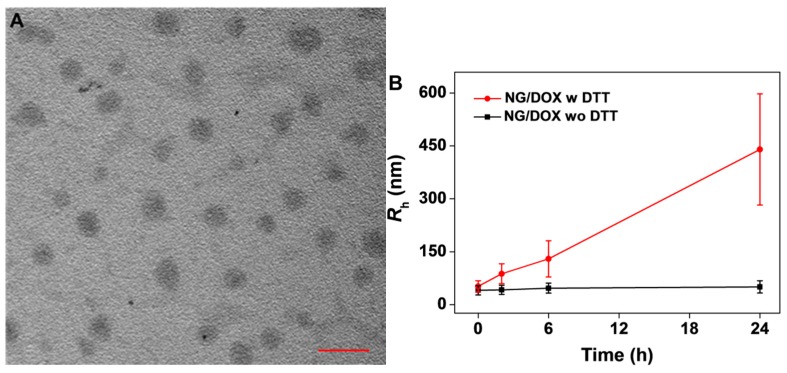

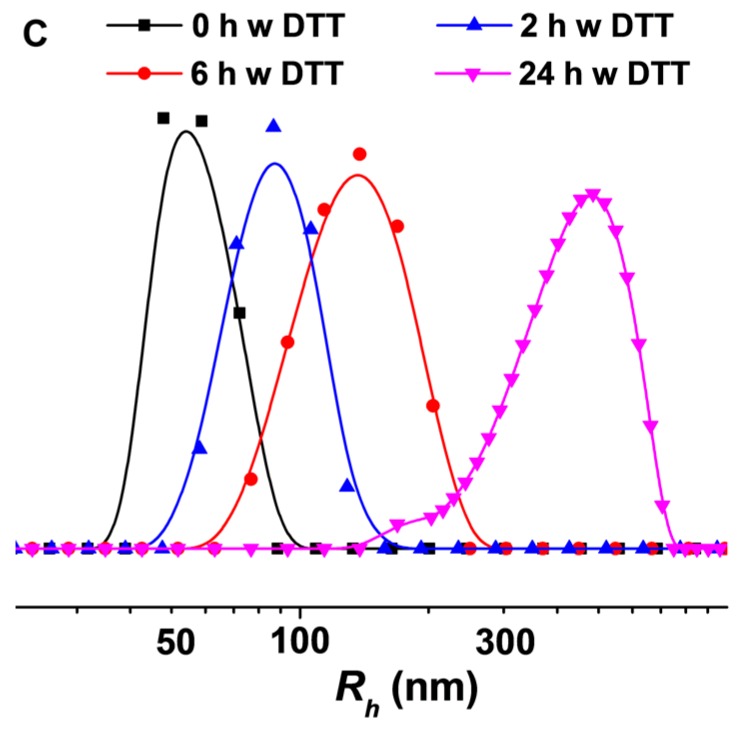
Typical TEM micrograph of NG/DOX dispersed in PBS at pH 7.4 (**A**). Scale bar meant 200 nm. DLS data changes of *R*_h_ (**B**) and distribution (**C**) of NG/DOX *versus* time in 24 h with or without 10.0 mM DTT. “w” represented “with”, and “wo” meant “without”.

As shown in Figure 2A, the TEM micrograph showed that NG/DOX exhibited a spherical morphology, and the visual diameters were 98 ± 5 nm. Meanwhile, the hydrodynamic radius (*R*_h_) of NG/DOX measured by DLS was 52 ± 16 nm (polydispersity index (PDI) = 0.31; Figure 2B). The difference between the TEM and DLS results should be attributed to the dehydration of loading nanogel in the preparation process of TEM specimen. In addition, as shown in Figure 2B, the size change of NG/DOX dispersed in PBS with 10.0 mM DTT was monitored by DLS. DTT is a kind of strong electronating agent, which could diffuse into the core of nanogel and break the disulfide bond (−S−S−) into two mercapto groups (−SH) [28]. As show in Figure 2B, the *R*_h_s of swollen nanogel in DTT-containing PBS at 0, 2, 6, and 24 h were detected to be 52 ± 16 nm (PDI = 0.3), 88 ± 28 nm (PDI = 0.32), 130 ± 51 nm (PDI = 0.39), and 440 ± 158 nm (PDI = 0.36), respectively. Next, as depicted in Figure 2C, the *R*_h_ distribution of nanogel in different periods elevated as the extension of time. In summary, the size increase of NG/DOX under the stimulation of DTT indirectly confirmed the reduction-response characteristic of NG/DOX *in vitro*.

**Figure 3:**
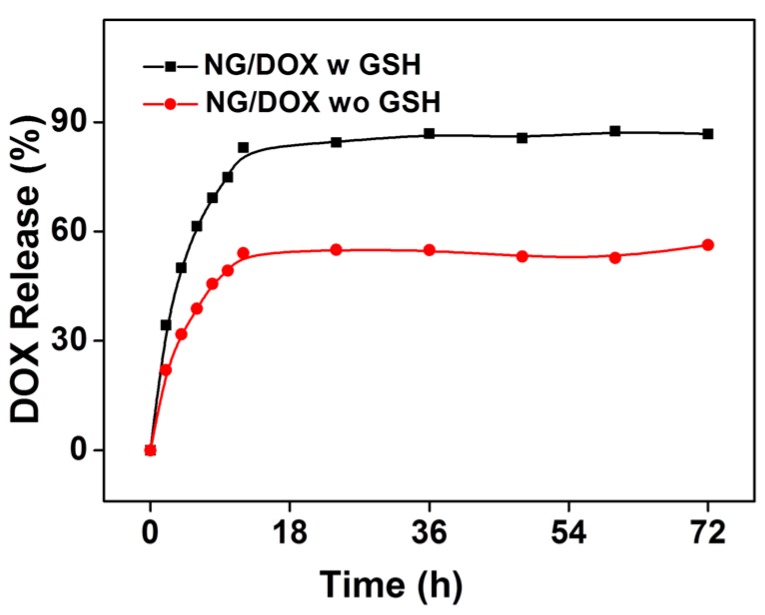
Release behavior of NG/DOX in PBS with or without 10.0 mM GSH at pH 7.4, 37 °C.

To determine the release behavior of NG/DOX and simultaneously demonstrate the reduction-responsive performance of NG/DOX, the *in vitro* DOX release profiles were assessed in PBS with or without GSH at pH 7.4, 37 °C with constant concussion of 70 rpm, mimicking the normal circulation condition in blood. As shown in Figure 3, the initial burst release was observed in PBS without GSH group. It was because the DOX molecule adhered to the surface of nanogel in the process of drug loading. As expected, 10.0 mM GSH could significantly accelerate the drug release from NG/DOX. After incubation for 12 h, the DOX release of both groups achieved balance, and the proportions of cumulative DOX release in NG/DOX w GSH and wo GSH groups were 83% and 54%, respectively. Then the platform period continued until 72 h. The reduction-response characteristic of NG/DOX should be attributed to the swelling of nanogel during the cleavage of the disulfide bond triggered by GSH. These profiles revealed that the nanogel not only reduced drug loss in circulation, but also enhanced the selective accumulation of the drug in tumor tissue by the EPR effect. As mentioned above, there was huge difference between the extracellular and intracellular reducibility; the smart polypeptide nanogel could directly release the payload in the tumor area and might achieve great success in clinical PCa chemotherapy.

### 3.2. Enhanced In Vitro Cellular Proliferation Inhibition and Accelerated Intracellular DOX Release of NG/DOX

**Figure 4:**
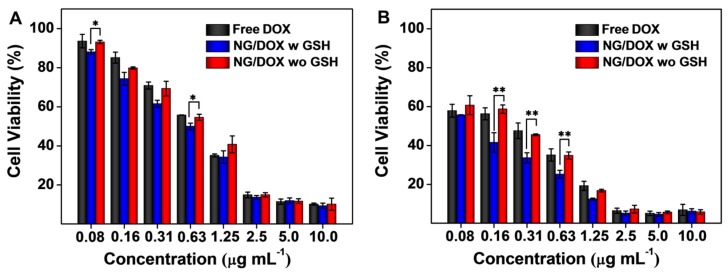
*In vitro* viability of RM-1 cells with or without 10.0 mM GSH pretreatment after co-incubation with free DOX and NG/DOX for 24 (**A**) and 48 h (**B**). Each set of data was represented as mean ± SD (*n* = 3; * *p* < 0.05, ** *p* < 0.01).

The *in vitro* proliferation inhibition ability of NG/DOX was assessed by a standard MTT assay. The results of 24 and 48 h MTT assays are shown in Figure 4. The 24 h half maximal inhibitory concentrations (IC_50_s) of free DOX and NG/DOX with or without the pretreatment of 10.0 mM GSH were calculated to be 0.79, 0.59, and 0.76 μg·mL^−1^, respectively. The 48 h IC_50_s of free DOX and NG/DOX with or without the pretreatment of 10.0 mM GSH were calculated to be 0.24, 0.07, and 0.23 μg·mL^−1^, respectively. As expected, the cytotoxicity of NG/DOX was enhanced by the GSH pretreatment *in vitro*. The IC_50_ of NG/DOX with 10.0 mM GSH pretreated in both 24 and 48 h were the lowest ones compared to those of free DOX and NG/DOX without the GSH pretreatment. In detail, the IC_50_ of NG/DOX with 10.0 mM GSH pretreated in 24 and 48 h were 0.8- and 0.3-fold lower than that of NG/DOX without the 10.0 mM GSH pretreatment, respectively. The difference of IC_50_s confirmed the reduction-response characteristic of the nanogel quantitatively and revealed that NG/DOX could take advantage of the redox potential between extracellular and intracellular conditions to promote antitumor efficacy. Interestingly, the cellular proliferation inhibition of NG/DOX without the GSH pretreatment was also better than that of free DOX. This phenomenon perhaps occurred because the tumor cells swallowed NG/DOX by pinocytosis, following by an efficient intracellular DOX release with the presence of GSH [29]. The controllable release property of NG/DOX enabled drug concentration to remain at a high level during the whole incubation period, resulting in more effective cytotoxic effect of NG/DOX than free DOX.

**Figure 5:**
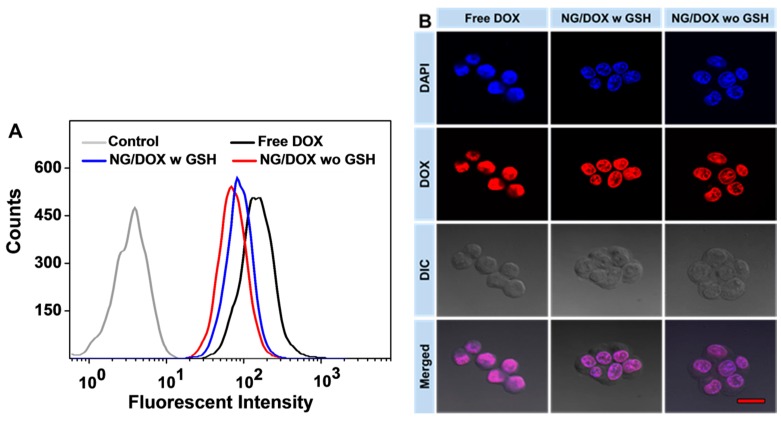
Typical FCM (**A**) and CLSM determinations (**B**) of toward RM-1 cells with or without 10.0 mM GSH pretreatment co-incubated with free DOX and NG/DOX for 2 h. Scale bar meant 20.0 μm.

As mentioned above, free DOX and NG/DOX were uptaken by RM-1 cells through different approaches, that is, diffusion and endocytosis, respectively, which might result in the difference of proliferation inhibition *in vitro*. In order to define the cellular uptake of free DOX and NG/DOX, FCM and CLSM were employed. In both the FCM and CLSM detections, RM-1 cells with or without the GSH pretreatment were incubated with free DOX or NG/DOX containing 5.0 μg·mL^−^^1^ equivalent DOX for 2 h. First, the FCM histograms of free DOX and NG/DOX are shown in Figure 5A. RM-1 cells without any treatment were used as control. The fluorescence intensities of DOX in the three groups were ranked as: free DOX > NG/DOX w GSH > NG/DOX wo GSH. This phenomenon might be attributed to the different cellular uptake approaches for free DOX and NG/DOX, and the accelerated DOX release from NG/DOX in the presence of GSH. Furthermore, the results of CLSM were shown in Figure 5B, the nuclei of RM-1 cells were dyed blue by DAPI, and DOX showed red fluorescence [8]. As expected, the CLSM microimages were fitted with the results of FCM fluorescence signal intensity. In a brief, both the FCM and CLSM results confirmed the efficient endocytosis and intracellular microenvironment-accelerated DOX release of NG/DOX, which was consistent with the GSH-accelerated release characteristic of NG/DOX *in vitro*.

### 3.3. Upregulated In Vivo Efficacy and Security of NG/DOX

To illustrate the advantages of NG/DOX for PCa treatment *in vivo*, the antitumor efficacy and security of free DOX and NG/DOX were tested against an RM-1 PCa-grafted C57BL/6 mouse model. Since the average tumor weight reached around 87.0 mg, the tumor-bearing mice were treated with NS, free DOX, and NG/DOX with 5.0 mg·kg^−1^ DOX equipment at 2, 4, and 9 days to mimic the clinical megadose chemotherapy. The changes in tumor weight, body weight, and survival rate were monitored every day after the start of treatment.

**Figure 6:**
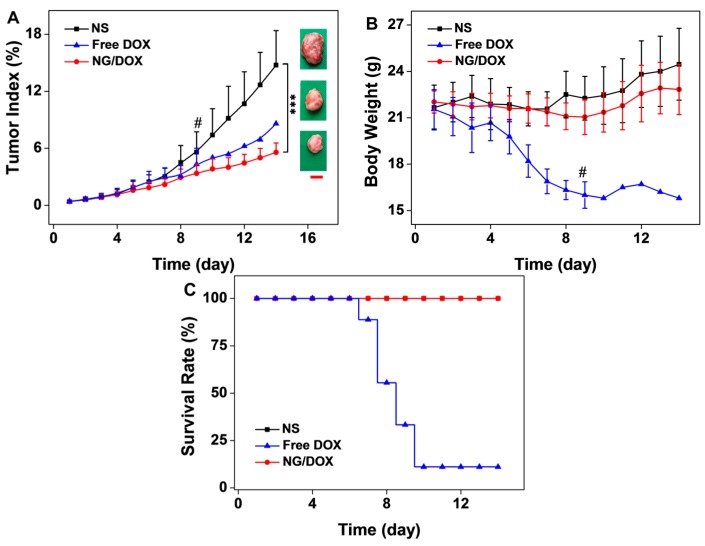
*In vivo* antitumor efficacies (**A**), body weight changes (**B**), and survival rates (**C**) of RM-1 PCa-grafted mice with treatments of NS as control, and free DOX and NG/DOX at a dose of 5.0 mg (kg·BW)^−1^ on Day 2, 4, and 9. ^#^ meant the mice widely dead in the free DOX group after the second treatment attributed to the frequent and megadose treatment. After the third treatment, just one mouse survived in the free DOX group. The tumor photos after all treatments were obtained and showed in the right inset of Figure 6A. Scale bar represented 2.0 cm. The data were represented as mean ± SD (*n* = 8; *** *p* < 0.001).

As shown in Figure 6A, the tumor indices during treatments and tumor photos after all treatments confirmed that both free DOX and NG/DOX were effective in inhibiting tumor growth compared with NS as control. Amazingly, compared to NS and free DOX, NG/DOX significantly blocked the increase of tumor index, showing great antitumor efficacy. On Day 14, the tumor index of NG/DOX was 5.6%, which was significantly less than those of NS (14.7%) and free DOX (8.6%). The improved tumor inhibition capability of NG/DOX should be attributed to the EPR effect and the reduction-response characteristic of l-cystine-crosslinked polypeptide nanogel. The loss of body weight was the symbolic information for evaluating the DOX-induced toxicity. In Figure 6B, the body weights were recorded every day after the first treatment. Before the first treatment, the average body weight of tumor-bearing mice was 21.8 g, and there was no significant difference in body weight among all the three groups. Although the mice treated with NG/DOX exhibited an average 5.62% decrease of body weight, the mice treated with free DOX lost more than 20% body weight. Unfortunately, most of the mice in the free DOX group died due to the frequent and megadose treatment. As shown in Figure 6C, after the third injection on Day 9, there was just one mouse survived in the free DOX group. This sad result may attribute to the frequent and high dose intravenous injection of free DOX. In contrast, no member of the NG/DOX group died in the whole treatment period. The comparison strongly confirmed the safety of NG/DOX. The rapidly loss of body weight and largely number of dead mice in the free DOX group indicated the serious systematic toxicity of free DOX and pointed out the improved security of reduction-positive NG/DOX *in vivo*.

**Figure 7:**
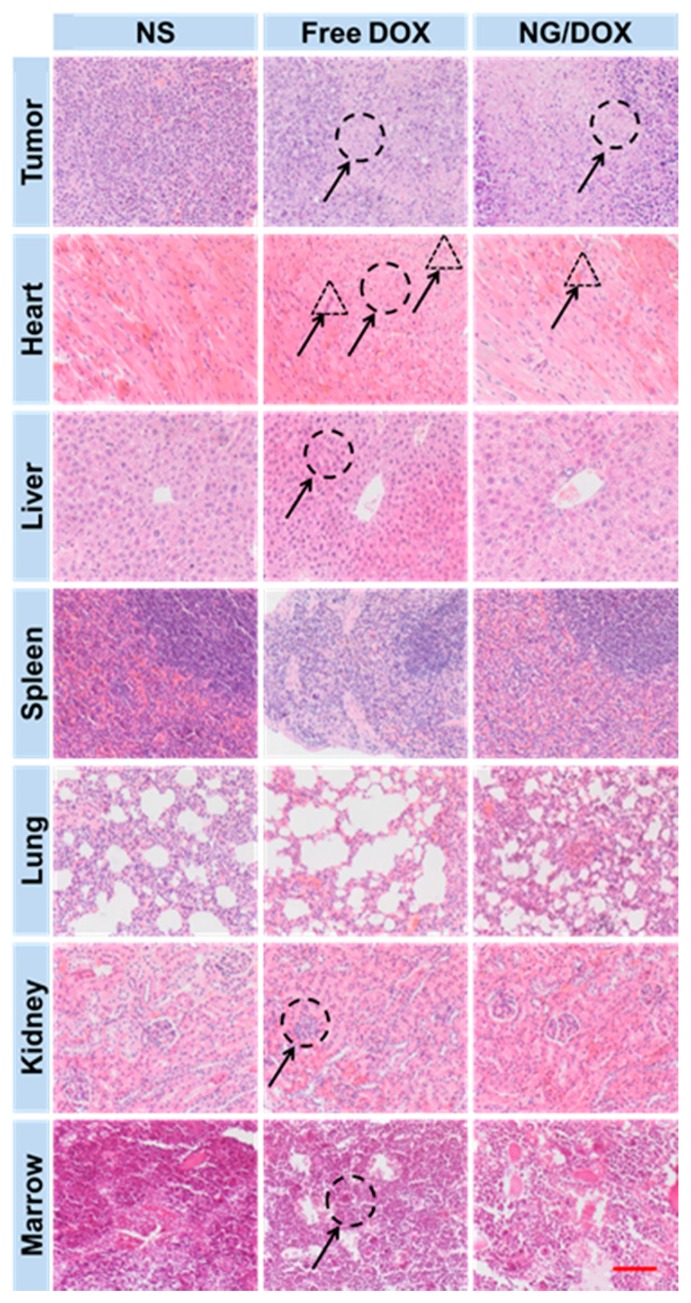
Histopathological analyses of tumors and visceral organ and tissue sections (*i.e.*, tumor, heart, liver, spleen, lung, kidney, and marrow) from RM-1 PCa-grafted mice after all treatments with NS, free DOX, and NG/DOX. The circles pointed out (i) the necrotic areas in tumors; (ii) the changes in cardiac muscle fibers; (iii) the pyknosis and shrinking of hepatocytes; (iv) the shrinking and abnormal glomeruli of kidneys; and (v) bone marrow cell micronuclei. The triangles point out the inflammatory cell infiltration in cardiac tissues in the free DOX and NG/DOX groups. Scale bar meant 200.0 μm.

Meanwhile, as depicted in Figure 7, the antitumor efficacy and systemic toxicity of both DOX formulations were further performed by the histopathological analyses of tumor and major organs (*i.e.*, heart, liver, spleen, lung, and kidney). Hematoxylin and eosin (H & E) staining is an important assay to evaluate tumor apoptosis and organ damage. The nuclei were stained dark blue by hematoxylin, while the cytoplasm and extracellular matrix were dyed red or pink by eosin. These qualitative results displayed the visual changes of tumors and major organs.

**Figure 8:**
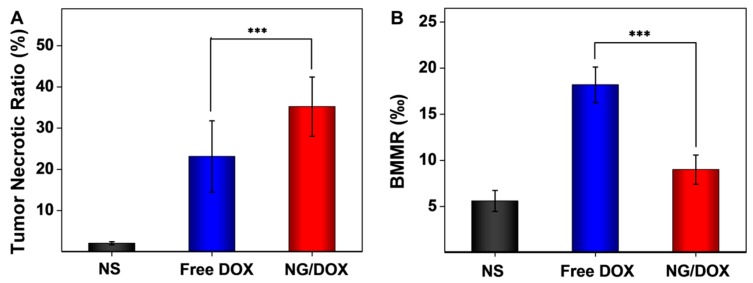
Semiquantitative analyses of tumor necrosis (**A**) and bone marrow cell micronucleus (**B**) from H & E microimages. Each set of data was represented as mean ± SD (*n* = 3 for A and 5 for B, *** *p* < 0.001).

It was obvious that the tumor cells had clear spherical shapes and different nucleus sizes in the NS group (Figure 7). However, large numbers of karyorrhexis and karyolysis nuclei, and extensive areas of tumor necrosis were observed in both the free DOX and NG/DOX groups. In particular, the necrotic area in the NG/DOX group was larger than that in the free DOX group. Furthermore, the semiquantitative analyses of necrotic area in tumors were performed from the H & E results and showed in Figure 8A. As exhibited, the NG/DOX group showed the maximum tumor necrotic area (35.2%) than the NS (2.1%) and free DOX groups (23.15%). The results confirmed a better tumor inhibitory effect from NG/DOX than that of free DOX, and showed great potential of the reduction-responsive nanogel for clinical use.

In clinical treatment, many antitumor drugs have serious side effects, so chemotherapy drugs always considered a double-edged sword for cancer patients. DOX, as one of the most commonly used chemotherapy drugs, always leads to severe organ damage after the intravenous injection of a large dose of the drug [30]. In this work, the histopathological changes of different organs caused by DOX were detected by H & E staining as shown in Figure 7. Free DOX rather than NG/DOX caused more damage to heart, liver, and kidney. Various types of heart damage, such as myocardial fiber breakage and inflammatory cell infiltration, were observed in the free DOX group. Compared to that of the free DOX group, the microimages from the NG/DOX group showed more regular cardiac muscle fiber arrangement and less inflammatory cell infiltration. In the H & E staining of different groups, liver lobes were exhibited as mentioned. Compared with those of the NS and NG/DOX groups, hepatocytes in the free DOX group were in coarctate order, and showed severe disturbance. The glomeruli in the free DOX group shrunk and lost the normal sphere shape. In contrast, the glomeruli in the NG/DOX and NS groups kept their normal sphere shape. In addition, the H & E staining microimages of lung and spleen suggested no difference caused by free DOX and NG/DOX. Consequently, the presence of smart nanogel in the formulation of NG/DOX significantly reduced the systemic toxicity.

To further confirm the safety of NG/DOX *in vivo*, the BMMR changes of all groups were detected. The chemotherapy drugs, *e.g.*, DOX, always cause loss or damage of chromosome, and induce the accumulation of bone marrow cells [15]. For this reason, cell micronucleus existed in bone marrow was a symbol of chemotherapy drug toxicity. As shown in Figure 8B, BMMRs were observed and analyzed in the H & E-stained sternum sections from the NS, free DOX, and NG/DOX groups. The quantity of the BMMR in each group was recorded: 5.6‰ for NS, 18.2‰ for free DOX, and 9.0‰ for NG/DOX. Compared to those of the NS and NG/DOX groups, the BMMRs from the RM-1 PCa-grafted mice with free DOX treatment was significantly elevated. The results revealed that DOX could induce the accumulation of bone marrow cells *in vivo*, and the reduction-responsive NG/DOX tremendously reduced the BMMR amount. In summary, the control delivery system assisted by the reduction-responsive polymer nanogel could down-regulate the injury caused by chemotherapy drug.

## 4. Conclusions

Herein, an l-cystine-crosslinked reduction-responsive polypeptide nanogel was employed as an efficient excipient for improved efficacy and reduced side effects of PCa chemotherapy both *in vitro* and *in vivo*. In detail, the reduction-responsive nanogel embedded with DOX could unload cargo rapidly in the mimicking intracellular microenvironment *in vitro*, and NG/DOX exhibited more antitumor efficacy and safety compared to free DOX *in vivo*. Therefore, the nanogel-based drug delivery system showed great potential for clinical chemotherapy. Although the nanogel exhibits fascinating merits for cancer chemotherapy, it still needs progress to improve the antitumor efficacy both *in vitro* and *in vivo*. For example, polymeric nanogel with tumor-specific targeting modification might achieve great success in the future.

## References

[1] Torre L.A., Bray F., Siegel R.L., Ferlay J., Lortet-Tieulent J., Jemal A. (2015). Global cancer statistics, 2012. CA Cancer J. Clin..

[2] Diaz M., Peabody J., Kapoor V., Sammon J., Rogers C., Stricker H., Lane Z., Gupta N., Bhandari M., Menon M. (2015). Oncologic outcomes at 10 years following robotic radical prostatectomy. Eur. Urol..

[3] Olsson C.E., Alsadius D., Pettersson N., Tucker S.L., Wilderang U., Johansson K.A., Steineck G. (2015). Patient-reported sexual toxicity after radiation therapy in long-term prostate cancer survivors. Br. J. Cancer.

[4] Katzenwadel A., Wolf P. (2015). Androgen deprivation of prostate cancer: Leading to a therapeutic dead end. Cancer Lett..

[5] Ding J., Xiao C., Li Y., Cheng Y., Wang N., He C., Zhuang X., Zhu X., Chen X. (2013). Efficacious hepatoma-targeted nanomedicine self-assembled from galactopeptide and doxorubicin driven by two-stage physical interactions. J. Control. Release.

[6] Xu W., Ding J., Xiao C., Li L., Zhuang X., Chen X. (2015). Versatile preparation of intracellular-acidity-sensitive oxime-linked polysaccharide-doxorubicin conjugate for malignancy therapeutic. Biomaterials.

[7] Perez-Herrero E., Fernandez-Medarde A. (2015). Advanced targeted therapies in cancer: Drug nanocarriers, the future of chemotherapy. Eur. J. Pharm. Biopharm..

[8] Wang J., Xu W., Ding J., Lu S., Wang X., Wang C., Chen X. (2015). Cholesterol-enhanced polylactide-based stereocomplex micelle for effective delivery of doxorubicin. Materials.

[9] Wang J., Xu W., Guo H., Ding J., Chen J., Guan J., Wang C. (2015). Selective intracellular drug delivery from pH-responsive polyion complex micelle for enhanced malignancy suppression *in vivo*. Colloids Surf. B Biointerfaces.

[10] Wang J., Shen K., Xu W., Ding J., Wang X., Liu T., Wang C., Chen X. (2015). Stereocomplex micelle from nonlinear enantiomeric copolymers efficiently transports antineoplastic drug. Nanoscale Res. Lett..

[11] Ding J., Xiao C., He C., Li M., Li D., Zhuang X., Chen X. (2011). Facile preparation of a cationic poly(amino acid) vesicle for potential drug and gene co-delivery. Nanotechnology.

[12] Ding J., Xiao C., Zhuang X., He C., Chen X. (2012). Direct formation of cationic polypeptide vesicle as potential carrier for drug and gene. Mater. Lett..

[13] Ding J., Zhuang X., Xiao C., Cheng Y., Zhao L., He C., Tang Z., Chen X. (2011). Preparation of photo-cross-linked pH-responsive polypeptide nanogels as potential carriers for controlled drug delivery. J. Mater. Chem..

[14] Ding J., Xu W., Zhang Y., Sun D., Xiao C., Liu D., Zhu X., Chen X. (2013). Self-reinforced endocytoses of smart polypeptide nanogels for “on-demand“ drug delivery. J. Control. Release.

[15] Huang K., Shi B., Xu W., Ding J., Yang Y., Liu H., Zhuang X., Chen X. (2015). Reduction-responsive polypeptide nanogel delivers antitumor drug for improved efficacy and safety. Acta Biomater..

[16] Ganju A., Yallapu M.M., Khan S., Behrman S.W., Chauhan S.C., Jaggi M. (2014). Nanoways to overcome docetaxel resistance in prostate cancer. Drug Resist. Updat..

[17] Zhang T., Xue X., He D., Hsieh J.T. (2015). A prostate cancer-targeted polyarginine-disulfide linked PEI nanocarrier for delivery of microRNA. Cancer Lett..

[18] Peppicelli S., Bianchini F., Calorini L. (2014). Extracellular acidity, a “reappreciated“ trait of tumor environment driving malignancy: Perspectives in diagnosis and therapy. Cancer Metastasis Rev..

[19] Gao Y., Zhou Y., Zhao L., Zhang C., Li Y., Li J., Li X., Liu Y. (2015). Enhanced antitumor efficacy by cyclic RGDyK-conjugated and paclitaxel-loaded pH-responsive polymeric micelles. Acta Biomater..

[20] Zheng X., Wang X., Mao H., Wu W., Liu B., Jiang X. (2015). Hypoxia-specific ultrasensitive detection of tumours and cancer cells *in vivo*. Nat. Commun..

[21] Zhao X., Liu P. (2015). Reduction-responsive core-shell-corona micelles based on triblock copolymers: Novel synthetic strategy, characterization, and application as a tumor microenvironment-responsive drug delivery system. ACS Appl. Mater. Interfaces.

[22] Gauthier M. (2014). Redox-responsive drug delivery. Antioxid. Redox Signal..

[23] Miao L., Holley A.K., Zhao Y., st Clair W.H., st Clair D.K. (2014). Redox-mediated and ionizing-radiation-induced inflammatory mediators in prostate cancer development and treatment. Antioxid. Redox Signal..

[24] Chaiswing L., Bourdeau-Heller J.M., Zhong W., Oberley T.D. (2007). Characterization of redox state of two human prostate carcinoma cell lines with different degrees of aggressiveness. Free Radic. Biol. Med..

[25] Ding J., Shi F., Xiao C., Lin L., Chen L., He C., Zhuang X., Chen X. (2011). One-step preparation of reduction-responsive poly(ethylene glycol)-poly(amino acid)s nanogels as efficient intracellular drug delivery platforms. Polym. Chem..

[26] Liu X., Wang J., Xu W., Ding J., Shi B., Huang K., Zhuang X., Chen X. (2015). Glutathione-degradable drug-loaded nanogel effectively and securely suppresses hepatoma in mouse model. Int. J. Nanomed..

[27] Leonetti C., D’Agnano I., Lozupone F., Valentini A., Geiser T., Zon G., Calabretta B., Citro G., Gabriella Z. (1996). Antitumor effect of c-myc antisense phosphorothioate oligodeoxynucleotides on human melanoma cells *in vitro* and in mice. J. Natl. Cancer Inst..

[28] Li Z., Zhang C., Wang B., Wang H., Chen X., Moehwald H., Cui X. (2014). Sonochemical fabrication of dual-targeted redox-responsive smart microcarriers. Acs Appl. Mater. Interfaces.

[29] Jeetah R., Bhaw-Luximon A., Jhurry D. (2014). Polymeric nanomicelles for sustained delivery of anti-cancer drugs. Mutat. Res..

[30] Li M., Tang Z., Lin J., Zhang Y., Lv S., Song W., Huang Y., Chen X. (2014). Synergistic antitumor effects of doxorubicin-loaded carboxymethyl cellulose nanoparticle in combination with endostar for effective treatment of non-small-cell lung cancer. Adv. Healthc. Mater..

